# Ce^3+^-ion-induced visible-light photocatalytic degradation and electrochemical activity of ZnO/CeO_2_ nanocomposite

**DOI:** 10.1038/srep31641

**Published:** 2016-08-16

**Authors:** Saravanan Rajendran, Mohammad Mansoob Khan, F. Gracia, Jiaqian Qin, Vinod Kumar Gupta, Stephen Arumainathan

**Affiliations:** 1 Department of Chemical Engineering and Biotechnology, University of Chile, Beauchef 850, Santiago, Chile; 2 Chemical Sciences, Faculty of Science, Universiti Brunei Darussalam, Jalan Tungku Link, Gadong, BE 1410, Brunei Darussalam; 3 Metallurgy and Materials Science Research Institute, Chulalongkorn University, Bangkok 10330, Thailand; 4 Department of Applied Chemistry, University of Johannesburg, Johannesburg, South Africa; 5 Department of Nuclear Physics, University of Madras, Guindy Campus, Chennai, 600 025, India

## Abstract

In this study, pure ZnO, CeO_2_ and ZnO/CeO_2_ nanocomposites were synthesized using a thermal decomposition method and subsequently characterized using different standard techniques. High-resolution X-ray photoelectron spectroscopy measurements confirmed the oxidation states and presence of Zn^2+^, Ce^4+^, Ce^3+^ and different bonded oxygen species in the nanocomposites. The prepared pure ZnO and CeO_2_ as well as the ZnO/CeO_2_ nanocomposites with various proportions of ZnO and CeO_2_ were tested for photocatalytic degradation of methyl orange, methylene blue and phenol under visible-light irradiation. The optimized and highly efficient ZnO/CeO_2_ (90:10) nanocomposite exhibited enhanced photocatalytic degradation performance for the degradation of methyl orange, methylene blue, and phenol as well as industrial textile effluent compared to ZnO, CeO_2_ and the other investigated nanocomposites. Moreover, the recycling results demonstrate that the ZnO/CeO_2_ (90:10) nanocomposite exhibited good stability and long-term durability. Furthermore, the prepared ZnO/CeO_2_ nanocomposites were used for the electrochemical detection of uric acid and ascorbic acid. The ZnO/CeO_2_ (90:10) nanocomposite also demonstrated the best detection, sensitivity and performance among the investigated materials in this application. These findings suggest that the synthesized ZnO/CeO_2_ (90:10) nanocomposite could be effectively used in various applications.

In recent years, many researchers have focused on semiconductor nanomaterials because of their versatile properties and applications^1^,^2^,^3^. Generally, such nanomaterials enable the highly sensitive detection of numerous enzymes and can degrade various organic pollutants^4^,^5^,^6^. Nanosemiconductor-based biosensing and photocatalysis are essential applications for monitoring, controlling and improving the conditions of the ecosystem^1^,^2^,^3^,^4^,^5^,^6^. Among the various semiconductors, zinc oxide (ZnO) is an efficient, non-toxic and low-cost material with a large band-gap energy (~3.2 eV). ZnO exhibits many fascinating properties and is used in multiple applications, including paints, sunscreens, solar cells, photocatalysts, antibacterial agents, biosensors, and gas sensors^7^,^8^,^9^,^10^,^11^.

Currently, textile dyes are the most important worldwide source of water pollution because of their release into water resources^7^. Water pollution is considered to be a major factor that affects the environment; hence, solving this issue will benefit human living conditions. In recent decades, many researchers have investigated the use of semiconductor nanometal oxides for the photocatalytic treatment of wastewater and other pollutants. This approach was believed to be the best way to reduce the contaminants present in the water under different light sources. The photocatalytic process of treating pollutants is the most advanced method because it requires a smaller amount of catalyst and generates almost no or very few harmful by-products^3^,^4^,^8^. Metal oxides are the most convenient materials used as photocatalysts for photocatalytic reactions because they have the capacity to eliminate the toxic contaminants present in industrial wastewaters^1^,^2^,^3^,^4^,^5^,^6^.

ZnO and cerium oxide (CeO_2_) are the predominant semiconductors used for the photocatalytic degradation of contaminated water^2^,^3^,^9^. However, the main disadvantage of both of these metal oxides is their limited photocatalytic activity under visible light because of their large band gaps. Therefore, substantial efforts have been devoted to developing hybrid photocatalysts that are active under visible light because the solar spectrum contains ~45% of visible light and is freely available. Generally, hybrid photocatalysts show improved performance compared to their respective individual components^10^,^11^. The achievement of visible-light photocatalysis depends on the prevention of electron-hole recombination^10^,^11^,^12^,^13^,^14^,^15^,^16^. The hindrance of electron-hole recombination is achieved through several methods, such as doping of metals (Fe, Au, Ag, Mn, and etc.,) or nonmetals (F, N, S and etc.,) into ZnO or forming ZnO-based composites such as ZnO/metal, ZnO/metal oxide and ZnO/polymer^10^,^11^,^12^,^13^,^14^,^15^,^16^. Among the different reported composites, the simplest and most viable composites are those that comprise different semiconductors, such as ZnO/CdO^13^, ZnO/Mn_2_O_3_^15^, and ZnO/CeO_2_^16^. Recently, we reported that ZnO nanorods combined with Mn_2_O_3_ can effectively prevent electron-hole recombination, and the resulting composite enabled the degradation of pollutants to be extended from the UV to the visible light range because of the synergetic effect between these two semiconductors^15^.

Conversely, nano-ZnO is well known and frequently used as an electrocatalyst to determine various enzymes. Ahmad *et al*. reported that ZnO nanosheets enabled the precise detection of uric acid (UA; 0.05–2 mM) because they provide a high electron density, which favors high sensitivity^17^. A glassy carbon electrode (GCE) with ZnO nanoflowers exhibited high sensitivity for the determination of dopamine because of the nanoflowers’ interesting hierarchical nanostructure^18^. Among several chemicals found in human beings, UA and ascorbic acid (AA) are critical constituents because they play a vital role in human health. Any deviation from the specific level of these constituents in the blood leads to dangerous diseases such as altered blood pressure, heart problems, hyperuricemia, gout, Lesch-Nyhan syndrome, arthritis and kidney stones^17^,^18^,^19^,^20^. Therefore, the precise determination of these constituents in the blood is important for clinical diagnoses during early stages of related diseases. Among the several analytical methods used for this purpose, electrochemical analysis has been demonstrated to be a very favorable approach for AA and UA detection.

To the best of our knowledge, no research studies have clearly elucidated the photocatalytic mechanism of ZnO/CeO_2_ nanocomposites for the degradation of organic pollutants and industrial textile effluents (real sample analysis) or the electrocatalytic mechanism by which ZnO/CeO_2_ nanocomposites detect AA and UA. Therefore, in the present study, ZnO, CeO_2_ and ZnO/CeO_2_ (99:01, 97:03, 95:05, 90:10, 80:20, 70:30, 60:40, 50:50 weight ratios) were synthesized using a simple thermal decomposition technique. The synthesized samples were characterized using different techniques, including X-ray diffraction (XRD), field-emission scanning electron microscopy (FE-SEM), high-resolution transmission electron microscopy (HR-TEM), high-resolution X-ray photoelectron spectroscopy (HR-XPS), UV-Vis spectrophotometry and Brunauer–Emmett–Teller (BET) analysis; the results are discussed in detail. All of the prepared materials were used for the photocatalytic degradation of methyl orange (MO), methylene blue (MB), phenol and industrial textile effluent (real sample analysis) under visible-light illumination. Additionally, the stability and reusability of the photocatalysts were investigated via recycling tests. Furthermore, the synthesized catalysts were also used as an electrochemical catalyst for the detection of UA and AA to determine the optimum ZnO/CeO_2_ catalyst composition. The optimized and highly sensitive ZnO/CeO_2_ (90:10) catalyst was finally used for the detection of various concentrations of UA and AA.

## Results and Discussion

**Figure 1:**
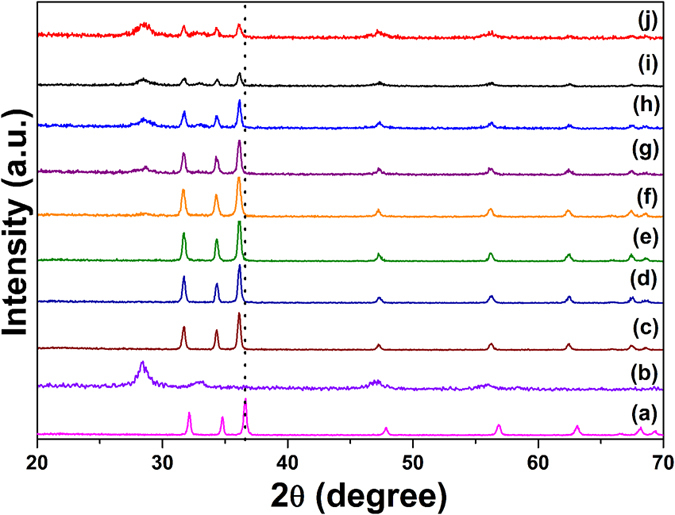
XRD patterns of (**a**) pure ZnO, (**b**) pure CeO_2_, (**c**) ZnO/CeO_2_ (99:01), (**d**) ZnO/CeO_2_ (97:03), (**e**) ZnO/CeO_2_ (95:05), (**f**) ZnO/CeO_2_ (90:10), (**g**) ZnO/CeO_2_ (80:20), (**h**) ZnO/CeO_2_ (70:30), (**i**) ZnO/CeO_2_ (60:40), and (**j**) ZnO/CeO_2_ (50:50) nanocomposites.

The structure of the prepared samples and their crystallite size were confirmed using powder XRD measurements. The diffraction patterns of ZnO/CeO_2_ nanocomposites with different component weight percentages were compared with those of pure ZnO and pure CeO_2_, as shown in Fig. 1. The XRD pattern of the pure ZnO is shown in Fig. 1(a). The results show that ZnO exhibits a hexagonal structure whose diffraction peaks correspond to *hkl* planes (100), (002), (101), (102), (110), (103) (200), (112) and (201); this pattern matches JCPDS No. 79-0208. Figure 1(b) shows the pattern of cubic-structured CeO_2_, whose peaks correspond to *hkl* planes (111), (200), (220) and (311); this pattern matches JCPDS No. 65-2975. The patterns of the ZnO/CeO_2_ nanocomposites with a CeO_2_ content of ≤5 wt% are shown in Fig. 1(c–e). The intensity of the CeO_2_ peaks in these patterns is very low, almost negligible. Therefore, we could not determine the lattice parameters of CeO_2_ from these XRD patterns. When the wt% of CeO_2_ in the nanocomposite was increased to 10 to 50 wt%, the intensities of the ZnO and CeO_2_ peaks decreased and increased, respectively (Fig. 1(f–j)). The variation of the intensity was due to the difference in scattering factors; i.e., the scattering factor of Ce^4+^ ions is greater than that of Zn^2+^ ions in the ZnO/CeO_2_ nanocomposites^21^. A similar observation was reported by Mishra *et al*.^21^. In Fig. 1(f–j), all of the diffraction patterns reveal the presence of two phases: hexagonal-structured ZnO and cubic-structured CeO_2_. The addition of CeO_2_ to the ZnO matrix does not affect the hexagonal structure of ZnO. The Scherrer formula was used to calculate the crystallite sizes of the ZnO/CeO_2_ nanocomposites on the basis of the (101) diffraction peak of ZnO and the (111) diffraction peak of CeO_2_. The calculated lattice parameters and the crystallite sizes are summarized in Table 1. Therefore, the XRD pattern confirmed the formation of ZnO/CeO_2_ nanocomposites without any impurities. The BET specific surface area values of the nanocomposites were in agreement with the XRD results; the values are tabulated in Table 1. Compared with the diffraction peaks of pure ZnO, those of the nanocomposite were slightly shifted to lower angles, confirming that many interfaces are present in the nanocomposites^22^.

**Figure 2:**
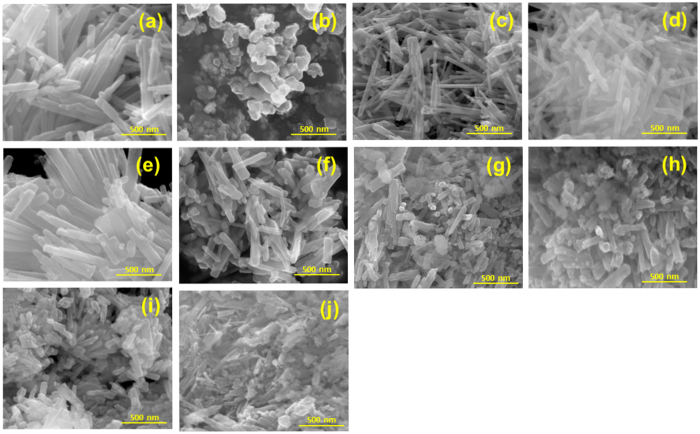
FE-SEM images of (**a**) ZnO, (**b**) CeO_2_, (**c**) ZnO/CeO_2_ (99:01), (**d**) ZnO/CeO_2_ (97:03), (**e**) ZnO/CeO_2_ (95:05), (**f**) ZnO/CeO_2_ (90:10), (**g**) ZnO/CeO_2_ (80:20), (**h**) ZnO/CeO_2_ (70:30), (**i**) ZnO/CeO_2_ (60:40), and (**j**) ZnO/CeO_2_ (50:50) nanocomposites.

The surface morphologies of the pure ZnO, pure CeO_2_ and ZnO/CeO_2_ (99:1, 97:3, 95:5, 90:10, 80:20, 70:30, 60:40 and 50:50) nanocomposites were confirmed using FE-SEM analysis; the corresponding images are shown in Fig. 2. Pure ZnO (Fig. 2(a)) was observed as irregular nanorods that were ~35 and ~500 nm in diameter and length, respectively. An FE-SEM image of the pure CeO_2_ sample is shown in Fig. 2(b); this image shows a large number of spherically shaped agglomerated nanoparticles because of the small particle size of the CeO_2_. The addition of CeO_2_ to ZnO appears to affect the morphology and size of the resulting nanocomposites, as clearly observed in the FE-SEM images (Fig. 2(c–j)). With increasing wt% of CeO_2_ in the nanocomposites, the size of the ZnO nanorods decreased. Furthermore, in cases where the CeO_2_ content was greater than 10 wt%, the nanocomposites consisted of highly agglomerated nanorods and spherically shaped particles. This simultaneous occurrence of nanorods and spherical particles might to attributable to the synergetic and nucleation effect between the ZnO and CeO_2_^23^,^24^,^25^. The composition of each element in the synthesized samples was confirmed and established using energy-dispersive X-ray spectroscopy (EDX) and are displayed in Fig. S1. The pure ZnO nanorods contained Zn and O, whereas pure CeO_2_ consisted of Ce and O. The 10 wt% and 30 wt% ZnO/CeO_2_ nanocomposites contained Zn, Ce and O, as clearly observed in Fig. S1(c) and (d). Therefore, the EDX spectra confirmed the synthesis of ZnO, CeO_2_ and ZnO/CeO_2_ samples and the absence of impurities.

**Figure 3:**
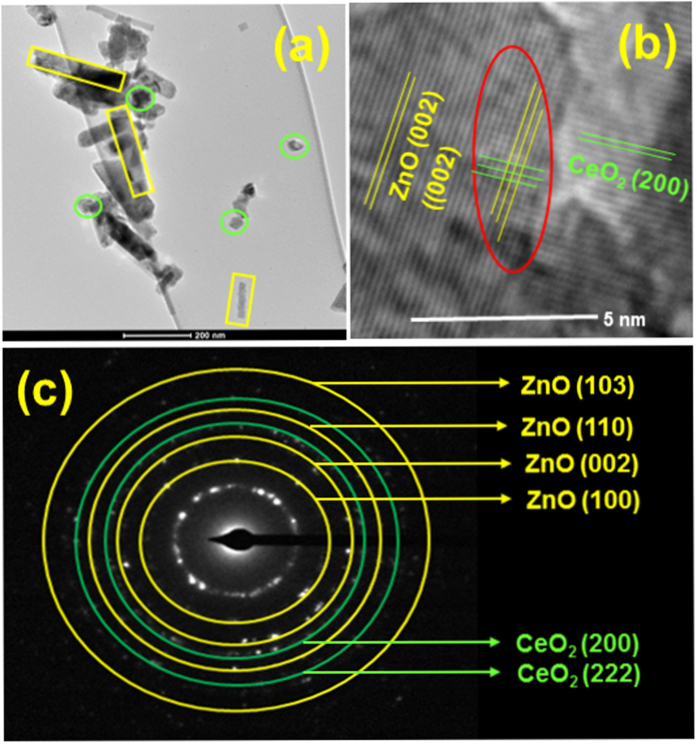
(**a**) TEM image, (**b**) HR-TEM image, and (**c**) SAED pattern of the ZnO/CeO_2_ (90:10) nanocomposite.

TEM and HR-TEM images of the ZnO/CeO_2_ (90:10) nanocomposite are shown in Fig. 3. The TEM image shows the ZnO nanorods along with CeO_2_ nanoparticles, which are highlighted in Fig. 3(a). The length of the ZnO nanorods was ~200 nm, and the diameter of the CeO_2_ nanoparticles was ~30 nm. The size of the ZnO nanorods decreased because of the CeO_2_ nanoparticles and the synergistic effect between CeO_2_ and ZnO^26^. The HR-TEM and selected-area electron diffraction (SAED) patterns further confirmed the structure of the materials. The HR-TEM image (Fig. 3(b)) clearly shows that, during the formation of the nanocomposite, synergetic and nucleation effects were induced between the ZnO and CeO_2_; in addition, defects were observed on the surface of the ZnO/CeO_2_ nanocomposite. The *d*-spacing values were calculated on the basis of the HR-TEM image and SAED pattern. The calculated *d*-spacing values show that the ZnO nanorods exhibited a hexagonal structure and that CeO_2_ exhibited a cubic structure, consistent with the XRD results.

The STEM micrographs and elemental mapping images for the nanocomposites are shown in Figs S2 and S3, respectively. To determine the location of ceria in the ZnO/CeO_2_ nanocomposites, samples were analyzed using STEM; the consistent bright- and dark-field images are shown in Fig. S2. Elemental mapping images based on these STEM images (highlighted square box) were collected (Fig. S3); the results clearly show that the nanocomposite contains Zn, Ce and O and that the CeO_2_ particles are dispersed uniformly rather than being separated individually or edged. These observations confirm that the CeO_2_ particles are located on the ZnO surface and are uniformly distributed.

**Figure 4:**
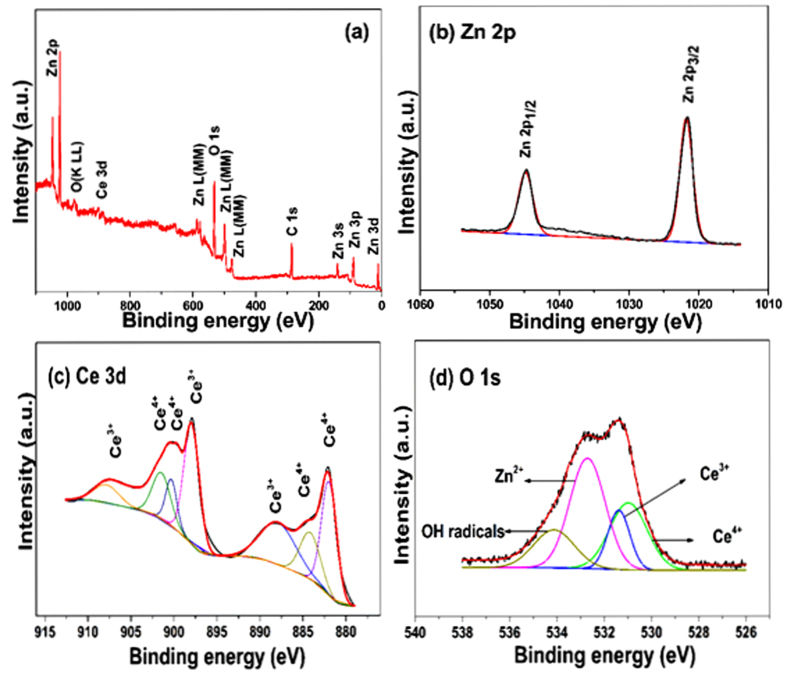
XPS spectra of ZnO/CeO_2_ (90:10) nanocomposite: (**a**) survey spectrum, (**b**) HR-XPS spectrum of Zn, (**c**) HR-XPS spectrum of Ce, and (**d**) HR-XPS spectrum of O 1 s.

The elemental composition and chemical states of the synthesized ZnO/CeO_2_ (90:10) nanocomposite were confirmed using XPS. The XPS survey spectrum is shown in Fig. 4(a). The ZnO/CeO_2_ surface is composed of only Zn, Ce, O, and C. The binding-energy values at 1021.6 eV and 1044.7 eV represent Zn 2p_3/2_ and Zn 2p_1/2_ in the ZnO/CeO_2_ nanocomposites, as shown in Fig. 4(b). This result indicates that the Zn exists in a Zn^2+^ oxidation state. The HR-XPS spectrum of the Ce peaks are presented in Fig. 4(c); their consistent binding energies represent the integrated peaks of Ce^3+^ and Ce^4+^ ions. Because of surface defects and a synergistic interaction between ZnO and CeO_2_, some Ce^3+^ sites were formed, which led to the formation of amorphous Ce_2_O_3_. Because the Ce_2_O_3_ was amorphous, it could not be detected by XRD, consistent with the XRD results in previous reports^2^,^3^. Four types of surface O were also observed (Fig. 4(d)) at binding energies of 532.6, 531.3, 530.8 and 534.1 eV; these different O binding energies are associated with Zn^2+^, Ce^3+^, Ce^4+^ and surface hydroxyl groups, respectively^2^,^3^,^27^. Therefore, the XPS results also confirmed the presence of Ce^3+^ in the ZnO/CeO_2_ nanocomposite.

**Figure 5:**
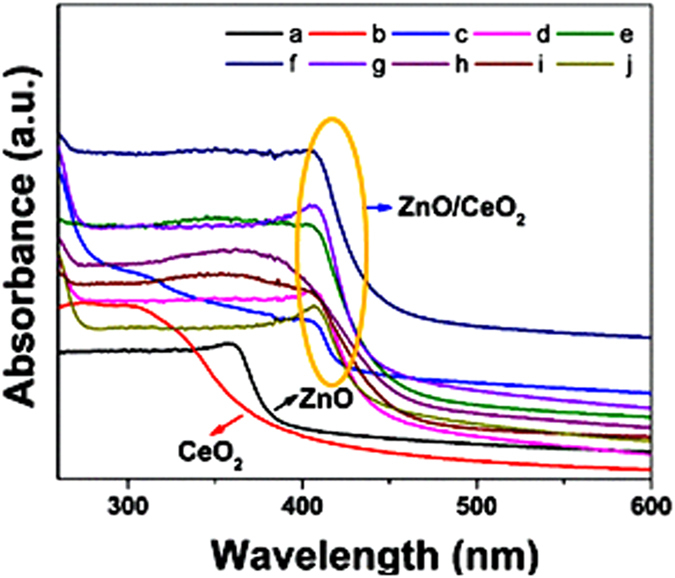
UV-vis absorption spectra of (**a**) ZnO, (**b**) CeO_2_, (**c**) ZnO/CeO_2_ (99:01), (**d**) ZnO/CeO_2_ (97:03), (**e**) ZnO/CeO_2_ (95:05), (**f**) ZnO/CeO_2_ (90:10), (**g**) ZnO/CeO_2_ (80:20), (**h**) ZnO/CeO_2_ (70:30), (**i**) ZnO/CeO_2_ (60:40), and, (**j**) ZnO/CeO_2_ (50:50) nanocomposites.

The estimation of the optical absorption wavelength of the prepared nanocomposites is an essential factor because, during photocatalysis, sufficient electrons will be excited from the valence band to the conduction band of the photocatalysts only if the energy of the incident light is equivalent to or greater than the photocatalysts’ band-gap energy. Otherwise, the photocatalytic activity will be limited or not occur^9^. Therefore, we measured the optical absorption wavelength of the synthesized ZnO/CeO_2_ nanocomposites (99:1, 97:3, 95:5, 90:10, 80:20, 70:30, 60:40 and 50:50), pure ZnO and pure CeO_2_ using a UV-vis spectrophotometer; the results are shown in Fig. 5. The absorption edge of the pure ZnO and CeO_2_ lies in the blue region; the corresponding wavelengths are ~388 nm (3.2 eV) and ~381 nm (3.25 eV), respectively, which lie in the UV region. Conversely, the ZnO/CeO_2_ nanocomposite showed (red shift) absorbance over a wider range (at wavelengths greater than 400 nm), which led to a shift toward the red region of the spectrum, as highlighted in Fig. 5. The band edge of the nanocomposite is wider because it contains amorphous Ce_2_O_3_, whereas the pure ZnO has a very sharp band edge^28^. This wider band edge for amorphous Ce_2_O_3_ has been speculated to arise from the formation of Ce^3+^ ions that have induced some localized mid-gap states in the band gap^2^,^3^. Therefore, the UV-vis absorption results confirm that the nanocomposite can harvest visible light and generate a greater number of electrons and holes under visible-light irradiation^15^. These results also suggest that, during the photocatalytic reactions, the generated holes and electrons could actively participate in oxidation and reduction reactions^15^.

### Photocatalytic degradation under visible-light illumination

**Figure 6:**
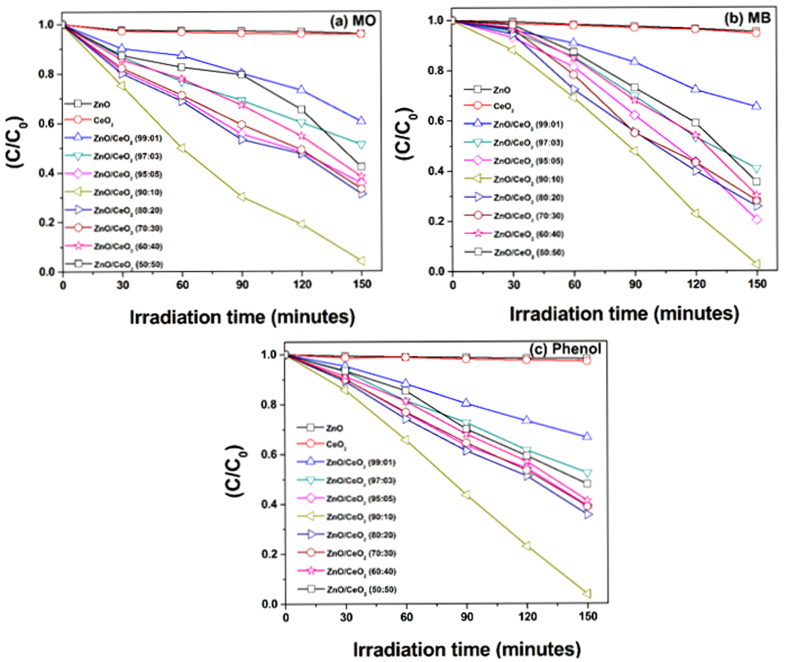
Time-dependent photocatalytic degradation curves of (**a**) MO, (**b**) MB, and, (**c**) phenol using all of the synthesized photocatalysts.

The photocatalytic activities of the synthesized pure ZnO, pure CeO_2_ and ZnO/CeO_2_ nanocomposites (99:1, 97:3, 95:5, 90:10, 80:20, 70:30, 60:40 and 50:50) were evaluated by degrading MO, MB and phenol under visible light irradiation. The degradation of MO, MB and phenol were evaluated by observing the change in the absorption of the initial concentration (C_0_) divided by the final concentration (C) of the dye as a function of the irradiation time (t); the corresponding results are shown in Fig. 6.

The degradation efficiency of all of the prepared photocatalysts were calculated; the results are tabulated in Table 2. The pure ZnO nanorods and the pure CeO_2_ nanospheres exhibited a reduced degradation efficiency because of their large band gaps^16^,^29^. Conversely, the ZnO/CeO_2_ nanocomposite exhibited a greater degradation efficiency because of its narrow band gap and wider absorption wavelength region, which resulted in the absorption of visible light and the production of more electrons and holes during the photocatalytic reaction. The degradation rate of MB was observed to be superior to those of MO and phenol. The greater degradation of MB is attributed to its structure; simultaneously, MB can function as a photocatalyst sensitizer^9^.

The first-order rate constant (k) was calculated using the formula k = ln(C/C_0_)/t, where C_0_ and C are the concentrations of MO, MB and phenol at the irradiation times 0 and t min, respectively. On the basis of this equation, the first-order rate constant (k) was determined; the results are tabulated in Table 2. The ZnO/CeO_2_ nanocomposite (90:10) exhibited a superior rate constant compared to those of pure ZnO, CeO_2_ and the other wt% nanocomposites because of its high surface area and the synergistic effect between CeO_2_ and ZnO^15^,^26^. The degradation results clearly show that the degradation efficiency does not increase monotonously with increasing CeO_2_ content beyond 10 wt%, which was considered the optimum weight percentage. CeO_2_ in concentrations up to the optimum amount acts as an electron–hole separation center and, hence, enhances the photocatalytic activity. However, when the CeO_2_ content is greater than 10 wt%, CeO_2_ begins to act as a charge-carrier recombination center and, hence, reduces the efficient separation of charge^11^,^30^. Consequently, we further investigated the best-performing degradation photocatalyst, ZnO/CeO_2_ (90:10), for the degradation of industrial textile effluent under visible-light irradiation.

**Figure 7:**
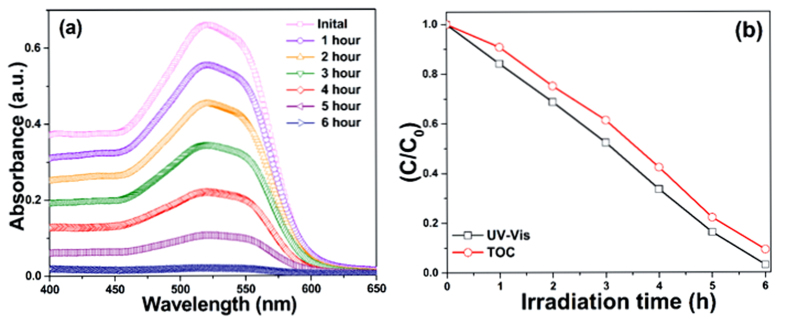
(**a**) Change in the absorption spectra of industrial textile effluent during photodecomposition, and (**b**) UV-vis and TOC plots indicating the degradation and mineralization of the industrial textile effluent using ZnO/CeO_2_ (90:10) photocatalyst for different exposure times under visible-light irradiation.

The procedure described elsewhere^26^ for photocatalytic decomposition of diluted industrial textile effluent was used in the present experiments. The photocatalytic decomposition of industrial textile effluent by the ZnO/CeO_2_ nanocomposite (90:10) is shown in Fig. 7(a) as a function of the irradiation time. These results clearly indicate that the intensity of the absorption band decreases with increasing irradiation time. The absorption spectrum appears to be completely flat after 6 h of irradiation, which confirms that almost all of the industrial textile effluent was decomposed by the ZnO/CeO_2_ (90:10) as a photocatalyst under visible-light irradiation.

The total organic carbon content (TOC) and UV-vis absorbance results (Fig. 7(b)) indicate that, with increasing irradiation time, the C/C_0_ value of the industrial textile effluent steadily decreases. This result demonstrates that, as the irradiation time increases, the concentration of the industrial textile effluent decreases. The TOC results confirm that ~90.2% degradation was achieved within 6 h.

**Figure 8:**
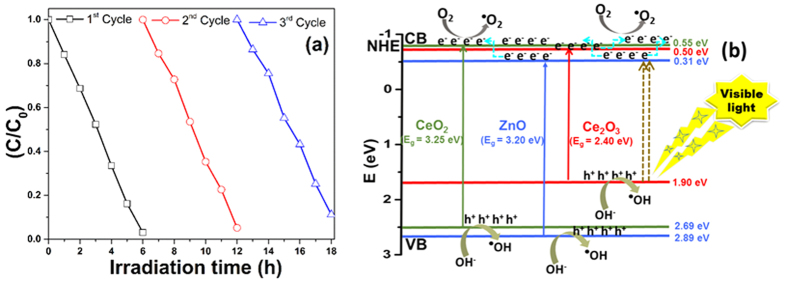
(**a**) Recycling performance and (**b**) schematic diagram representing the photocatalytic degradation mechanism of the ZnO/CeO_2_ (90:10) photocatalyst.

The stability and reusability analysis of the ZnO/CeO_2_ (90:10) photocatalyst was conducted using a recycling test; the results are shown in Fig. 8(a). After three recycling processes, the results of the industrial textile effluent degradation efficiency by the ZnO/CeO_2_ (90:10) photocatalyst were observed to have changed only slightly. Therefore, these results confirm that the ZnO/CeO_2_ (90:10) nanocomposite exhibits good efficiency and stability and can be used repeatedly in extended environmental applications.

Figure 8(b) is a schematic of the mechanism of the ZnO/CeO_2_ nanocomposite-catalyzed photocatalytic degradation of the pollutants under visible-light irradiation. The positions of the conduction and valence bands of the Ce_2_O_3_, CeO_2_ and ZnO were noted using the formula E_vb_ = X − E_e _+ 0.5E_g_, where X is the electronegativity of the Ce_2_O_3_, CeO_2_ and ZnO semiconductors calculated as the geometric mean of the electronegativity of the constituent atoms^26^. The electronegativity values for Ce_2_O_3_, CeO_2_ and ZnO were 5.20 eV, 5.57 eV and 5.79 eV, respectively^31^,^32^. E_e_ is the energy of the free electrons on the hydrogen scale (~4.5 eV), and E_g_ is the band-gap energy of Ce_2_O_3_ (2.4 eV standard value^32^), CeO_2_ (3.25 eV, as calculated on the basis of UV-vis absorption results) and ZnO (3.20 eV, as calculated on the basis of UV-vis absorption results) semiconductors. During the photocatalytic reaction, when the surface of the ZnO/CeO_2_ nanocomposite is irradiated with visible light, electrons in the valence band of Ce_2_O_3_ are excited because of its small band gap^2^,^33^. The excited electrons are transferred to the conduction band of ZnO. The conduction band edge positions of ZnO, CeO_2_ and Ce_2_O_3_ are very close to each other. The injection of electrons from the conduction band of ZnO into conduction band of CeO_2_ and Ce_2_O_3_ nanoparticles is expected to retard the reverse reaction between the photogenerated charge carriers^13^. At the same time, the transfer of electrons from the CeO_2_ to Ce_2_O_3_ also occurs. The electrons then react with and adsorb oxygen molecules to form superoxide radicals, which are responsible for the degradation/oxidation of the organic pollutants^2^. This oxidation process is capable of decomposing the industrial textile effluent and other pollutants under visible-light treatment. Moreover, the ZnO/CeO_2_ photocatalyst demonstrated a red shift in the absorption wavelength range (Fig. 5) and exhibited defects on its surface, which stimulates the formation of more electron and hole pairs during the irradiation and minimizes their recombination through interfacial transfer^34^. Additionally, ZnO/CeO_2_ nanocomposites have different energy states; therefore, during the photocatalytic reaction, the recombination of the photogenerated electrons and holes can be prevented, which is helpful in improving the photocatalytic activity of the photocatalyst under visible-light irradiation^34^.

In addition, another possible explanation for the high efficiency of the nanocomposite is the presence of an increased number of interfaces, which was confirmed by STEM and XRD. Yang *et al*. clearly explained that the interface region does not serve as a recombination center for the charge carriers and imparts the materials with greater stability, which in turn results in a highly efficient and stable photocatalyst because of the superior degradation within a short irradiation time^35^.

### Electrochemical studies

**Figure 9:**
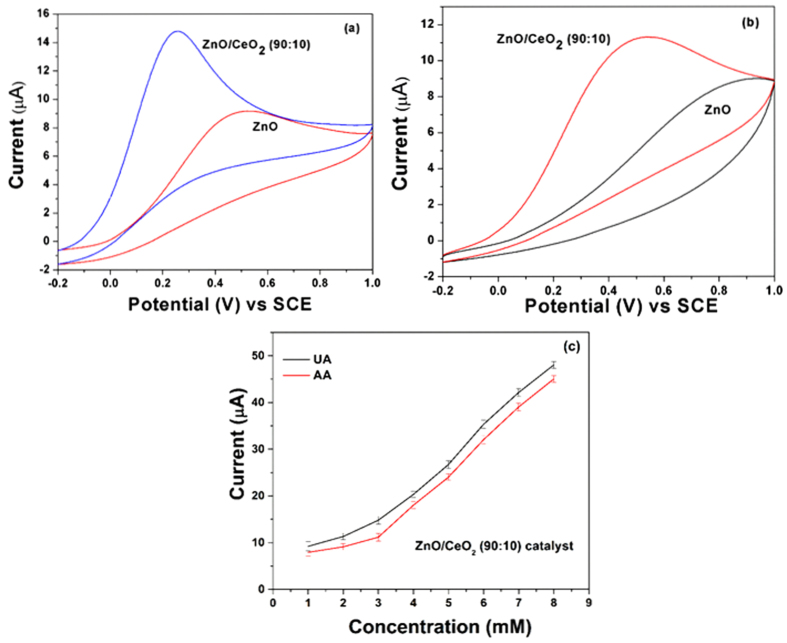
The CV results of pure ZnO nanorods and ZnO/CeO_2_ (90:10) nanocomposite in the presence of 3 mM of (**a**) UA, (**b**) AA, and (**c**) current response for various concentrations of UA and AA (1–8 mM) in the presence of the ZnO/CeO_2_ (90:10) nanocomposite.

Electrochemical experiments were performed to examine the sensing ability of the ZnO/CeO_2_ nanocomposite for UA and AA. The cyclic voltammetry (CV) responses of a bare GCE and GCEs modified with pure ZnO nanorods and ZnO/CeO_2_ nanocomposites were collected at a scan rate of 50 mV/s against 3 mM UA; the results are shown in Fig. S4. The results show that an increase in the CeO_2_ percentage also increases the current response at concentrations up to 10 wt%; however, at concentrations greater than 10 wt% CeO_2_, the current response decreases (Fig. S4). The 90:10 ZnO/CeO_2_ nanocomposite exhibited the highest sensitivity among the investigated materials (Fig. S4). The CV results for pure ZnO and ZnO/CeO_2_ (90:10) in the presence of 3 mM of UA and AA are illustrated in Fig. 9(a,b). These results also confirm that, compared to the pure ZnO nanorods, the ZnO/CeO_2_ (90:10) nanocomposite exhibits a higher current response at a lower potential.

The highly sensitive ZnO/CeO_2_ nanocomposite (90:10) was further used to detect various concentrations of UA and AA (1–8 mM); the results are shown in Fig. 9(c). Cobalt-doped hematite nanospheres have been previously reported to effectively detect UA and AA because of their large available surface area and small band gap^36^. Choi *et al*. reported that the morphology of the nanomaterials is also an important parameter for sensing; hence, less agglomerated Co_3_O_4_ nanoparticles exhibit enhanced sensing behavior compared to more agglomerated Co_3_O_4_ nanoparticles^37^. Similarly, in this case, ZnO/CeO_2_ (90:10) exhibits higher sensitivity compared to the other compositions and pure ZnO because of its lower extent of aggregation, higher surface area, and a smaller band gap. This finding also strongly coincides with the BET, FE-SEM and UV-vis spectroscopy results. Therefore, the high sensitivity of the ZnO/CeO_2_ nanocomposite toward UA and AA is due to its high surface area and reduced agglomeration.

## Conclusion

The ZnO/CeO_2_ nanocomposites were successfully synthesized using a simple thermal decomposition method and were characterized using standard techniques. The XPS results confirm the presence of Ce^3+^ ions in the ZnO/CeO_2_ nanocomposite. When compared with pure ZnO and CeO_2_ as well as all other compositions of ZnO/CeO_2_, the ZnO/CeO_2_ (90:10) nanocomposite exhibited superior photocatalytic degradation of pollutants and a high sensitivity for the electrochemical detection of AA and UA because of the presence of Ce^3+^ ions, a large available surface area and less agglomeration. Therefore, because of its promising and enhanced photocatalytic degradation performance and electrochemical detection capability, this ZnO/CeO_2_ (90:10) nanocomposite can be effectively used for future environmental and clinical applications.

## Experimental Procedure

### Materials

Zinc acetate dihydrate, cerium(III) acetate hydrate, AA, UA, MB, MO and phenol were purchased from Sigma-Aldrich. All aqueous solutions were prepared using double-distilled water.

### Synthesis of ZnO, CeO_2_ and ZnO/CeO_2_ nanocomposites

Preparation of the ZnO, CeO_2_ and ZnO/CeO_2_ nanocomposites was performed using the vapor-to-solid mechanism^38^. Increasing the temperature of the reactants (raw materials) resulted in the formation of vapors, which, upon cooling, settled/deposited onto a crucible. At the beginning of the condensation reaction, defects on the surface of the crucible (substrate) acted as nucleation sites for the oxide vapors^34^. Further condensation permitted such nuclei to grow into nanoparticles. On the basis of this mechanism, ZnO was prepared as follows: 3.0 g of zinc acetate dihydrate (raw material) was ground for more than 3 h using an agate pestle and mortar. The ground raw material was placed in an alumina crucible and calcined in a muffle furnace at 350 °C for 3 h. CeO_2_ nanomaterial was prepared in a similar manner using cerium(III) acetate hydrate as the raw material under the same conditions. ZnO/CeO_2_ nanocomposites with different weight percentages were prepared by mixing various weight percentages of zinc acetate dihydrate and cerium(III) acetate (in weight ratios of 99:1, 97:3, 95:5, 90:10, 80:20, 70:30, 60:40 and 50:50). The mixtures were ground for more than 3 h and calcined in a muffle furnace at 350 °C for 3 h.

### Photocatalytic experiment

The degradation of colored dyes as well as colorless pollutants is highly significant because wastewater contains many colored and colorless toxic chemicals^26^. Therefore, in this report, we selected two colored dyes (MO and MB) as well as colorless phenol as model pollutants. Initially, 500 mg of the photocatalyst was mixed with 500 mL of an aqueous pollutant solution (MO, MB and phenol) in a 600 mL cylindrical vessel. The vessel was covered by a 0.5% aqueous K_2_Cr_2_O_7_ solution circulating in a glass jacket to prevent UV radiation. The source of the visible light was a projection lamp (7748XHP 250 W, Philips) fitted in the photoreactor. The procedure used to prepare the dyes and other procedures were adopted from our previous reports^15^,^25^. On the basis of the results of the degradation experiments involving the model dyes and phenol, we determined that the ZnO/CeO_2_ nanocomposite (90:10) exhibited the greatest photocatalytic degradation activity. Furthermore, this optimized and highly efficient photocatalyst was used in subsequent degradation experiments involving industrial textile effluent (real sample analysis).

### Electrochemical experiments

All of the electrochemical measurements were performed on a PGSTAT-12 electrochemical work station (AUTOLAB, The Netherlands). The measurements were based on a three-electrode system with GCE (0.07 cm^2^) used as the working electrode, a Pt wire (~20 cm^2^) as the counter electrode and a saturated calomel electrode (SCE) as the reference electrode. Prior to each experiment, the GCE surface was polished with fine-grade alumina powder to a mirror finish, sonicated for approximately 15 min in double-distilled water, degreased with acetone and washed with an abundant amount of double-distilled water. All of the solutions were purged with nitrogen (99.99%) for 30 min before each electrochemical measurement, and a nitrogen environment was maintained throughout the experiments. Pure ZnO, CeO_2_ and ZnO/CeO_2_ were used to modify the GCE as follows: 3.0 mg of pure ZnO, pure CeO_2 _or ZnO/CeO_2_ nanocomposite was separately suspended in 3.0 mL of ethanol and subjected to ultrasonic agitation for 30 min to obtain a homogeneous suspension. A polished GCE was coated with 5 *μ*L of the prepared suspension to prepare GCEs modified with pure ZnO, pure CeO_2_ or ZnO/CeO_2_ nanocomposite.

### Characterization details

The structure and crystallite size of the prepared materials were determined using an X-ray diffractometer (Rich Seifert 3000, Germany) equipped with a Cu K_α1_ radiation source (λ = 1.5406 Å). The presence of the elements in the photocatalyst and their oxidation states were examined using XPS (DRA 400–XM1000 OMICRON, ESCA^+^, Omicron Nanotechnology, Germany). The specific surface area was calculated using the BET equation and data obtained using a Micromeritics ASAP 2020 (USA). The surface morphology, elemental analysis and EDX analysis were conducted using FE-SEM (HITACHI-SU6600, Hitachi, Japan). HR-TEM was performed using a Tecnai F20-FEI (USA). The optical properties and photocatalytic activities were measured using a UV-vis spectrophotometer (RX1, Perkin-Elmer, USA). Photocatalytic degradation of the industrial textile effluent was confirmed using the total organic carbon (TOC) content, which was measured using a TOC analyzer (Shimadzu, Japan).

## Additional Information

**How to cite this article**: Rajendran, S. *et al*. Ce^3+^-ion-induced visible-light photocatalytic degradation and electrochemical activity of ZnO/CeO_2_ nanocomposite. *Sci. Rep.* **6**, 31641; doi: 10.1038/srep31641 (2016).

## Supplementary Material

Supplementary Information

## Figures and Tables

**Table 1:** Lattice parameters, crystallite sizes (D) and BET surface areas of all of the prepared samples.

Sample	ZnO hexagonal 79-0208		CeO_2_ cubic 65-2975	ZnO D (nm)	CeO_2_ D (nm)	BET surface area (m^2^/g)
	a (Å)	c (Å)	a (Å)			
ZnO	3.262 (1)	5.206 (2)	—	35	—	8.6
CeO_2_	—	—	5.430(2)	—	9	26.2
ZnO/CeO_2_(1)	3.273 (9)	5.221 (4)	—	34	—	9.1
ZnO/CeO_2_(3)	3.268 (8)	5.220 (3)	—	33	—	9.5
ZnO/CeO_2_(5)	3.273 (9)	5.219 (3)	—	35	—	10.2
ZnO/CeO_2_(10)	3.270 (9)	5.215 (3)	5.411 (2)	29	6	13.2
ZnO/CeO_2_(20)	3.271 (9)	5.219 (3)	5.398 (2)	31	7	12.1
ZnO/CeO_2_(30)	3.266 (1)	5.215 (3)	5.403 (1)	32	8	11.5
ZnO/CeO_2_(40)	3.267 (3)	5.216 (3)	5.423 (2)	33	10	11.1
ZnO/CeO_2_(50)	3.262 (2)	5.220 (2)	5.413 (1)	35	11	10.3

**Table 2:** Degradation efficiency and first-order rate constant of all of the prepared photocatalysts.

Photocatalyst	MO degradation efficiency (%)	First-order rate constant (k) 10^−4 ^min^−1^	MB degradation efficiency (%)	First-order rate constant (k) 10^−4 ^min^−1^	Phenol degradation efficiency (%)	First-order rate constant (k) 10^−4 ^min^−1^
ZnO	4.0	0.0199	4.7	0.0201	1.9	0.0092
CeO_2_	4.2	0.0236	5.5	0.0233	2.9	0.0141
ZnO/CeO_2_(99:01)	39.5	0.1940	34.7	0.1760	33.5	0.1710
ZnO/CeO_2_(97:03)	48.8	0.2886	59.2	0.3413	47.7	0.2673
ZnO/CeO_2_(95:05)	64.4	0.4340	79.7	0.5333	60.4	0.3686
ZnO/CeO_2_(90:10)	95.9	1.1260	97.4	1.1046	96.2	1.0433
ZnO/CeO_2_(80:20)	68.8	0.4706	74.3	0.5233	64.4	0.4053
ZnO/CeO_2_(70:30)	66.8	0.4360	72.0	0.4860	61.1	0.3706
ZnO/CeO_2_(60:40)	61.8	0.3686	70.0	0.4040	58.8	0.3373
ZnO/CeO_2_(50:50)	57.9	0.2920	64.6	0.3446	52.2	0.2906
