# Supplementary material for: Ce3+-ion-induced visible-light photocatalytic degradation and electrochemical activity of ZnO/CeO2 nanocomposite
Source: Sci Rep. 2016 Aug 16;6:31641. doi: 10.1038/srep31641 (PMC4985814; doi:10.1038/srep31641)
Supplement: Supplementary Information [file srep31641-s1.doc]

Supporting Information

**Ce^3+^-ion-induced visible-light photocatalytic degradation and electrochemical activity of ZnO/CeO_2_ nanocomposite**

Saravanan. Rajendran,^a^* Mohammad Mansoob Khan,^b^ F. Gracia,^a^ Jiaqian Qin,^c^

Vinod Kumar Gupta,^d,e^ & Stephen Arumainathan,^f^**

*^a^Department of Chemical Engineering and Biotechnology, University of Chile, Beauchef 850, Santiago, Chile.*

*^b^Chemical Sciences, Faculty of Science, Universiti Brunei Darussalam, Jalan Tungku Link, Gadong, BE 1410, Brunei Darussalam.*

*^c^Metallurgy and Materials Science Research Institute, Chulalongkorn University, Bangkok 10330, Thailand*

*^d^Department of Chemistry, Indian Institute of Technology Roorkee, Roorkee, 247 667, India.*

*^e^Department of Applied Chemistry, University of Johannesburg, Johannesburg, South Africa*

*^f^Department of Nuclear Physics, University of Madras, Guindy Campus, Chennai, 600 025, India.*


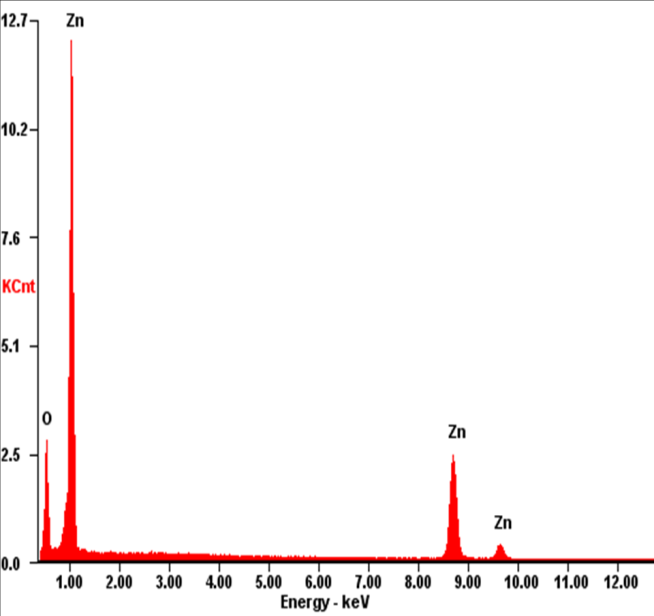

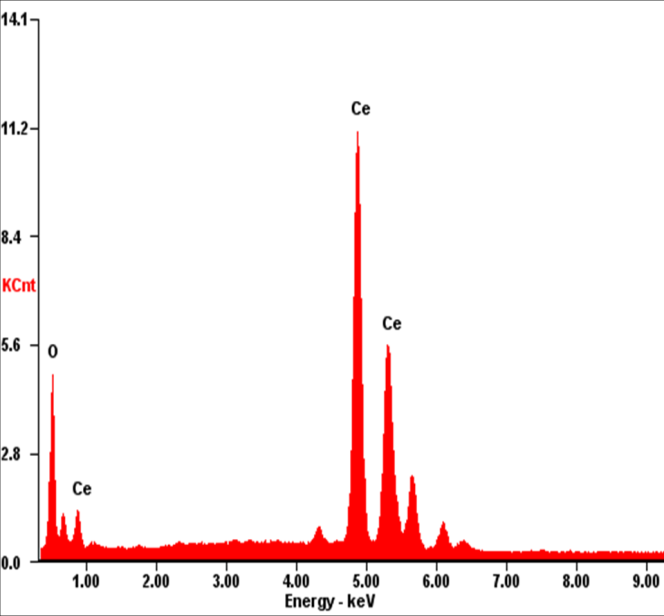

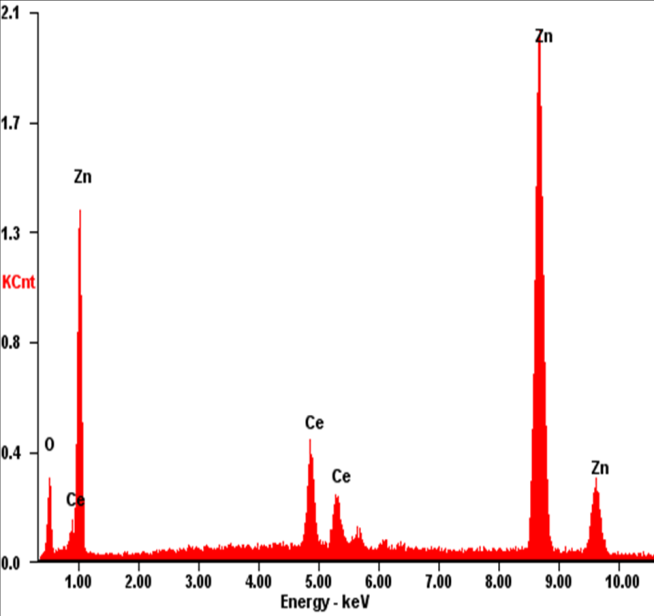

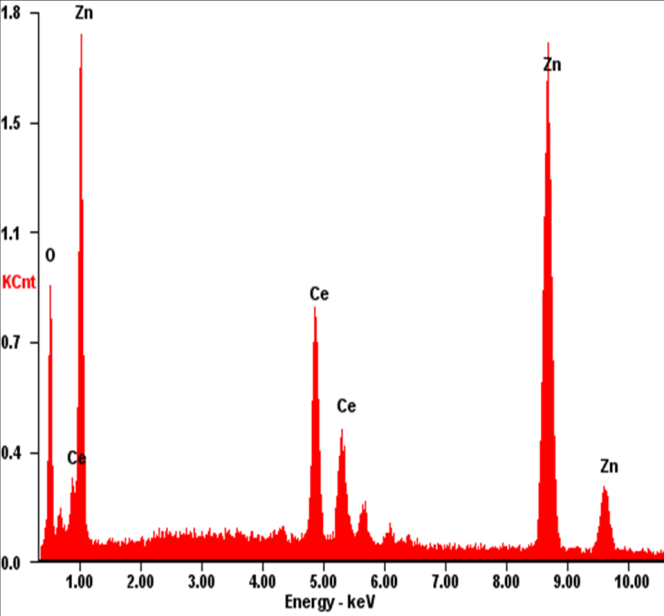


**(a)**

**(d)**

**(c)**

**(b)**

**Fig. S1.** EDS spectra of (**a**) ZnO, (**b**) CeO_2_, (**c**) ZnO/CeO_2_ (90:10), and, (**d**) ZnO/CeO_2_ (70:30).


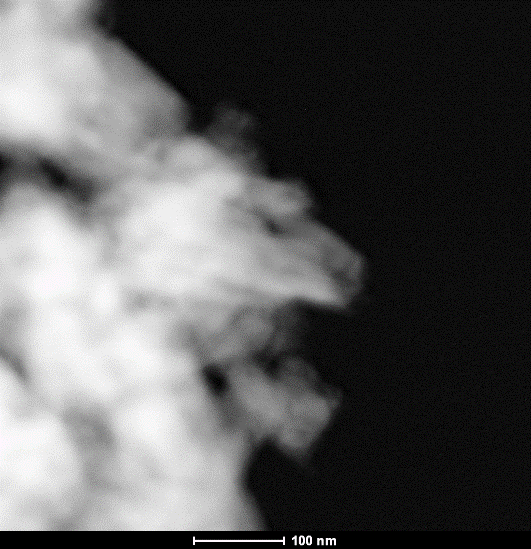

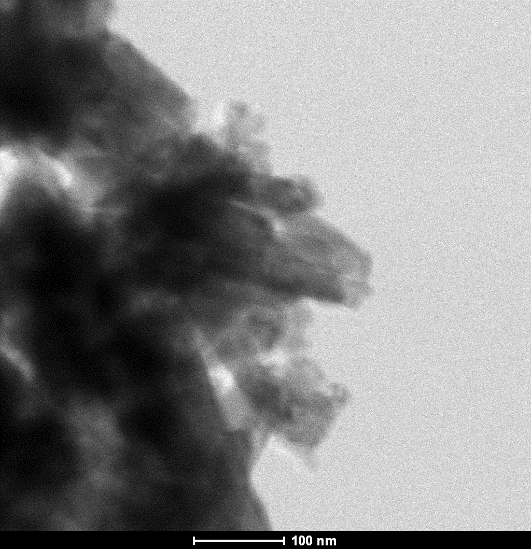


**Fig. S2.**  STEM bright and dark images of ZnO/CeO_2_ nanocomposite. The selected area shows the elemental mapping region.


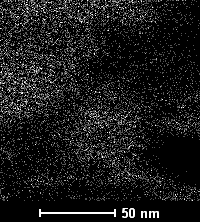

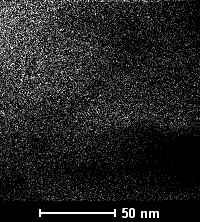

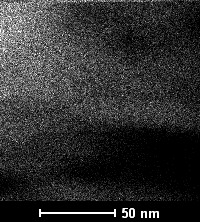


**Zn**

**Ce**

**O**

**Fig. S3.**  Elemental mapping of binary ZnO/CeO_2_ nanocomposite.

**
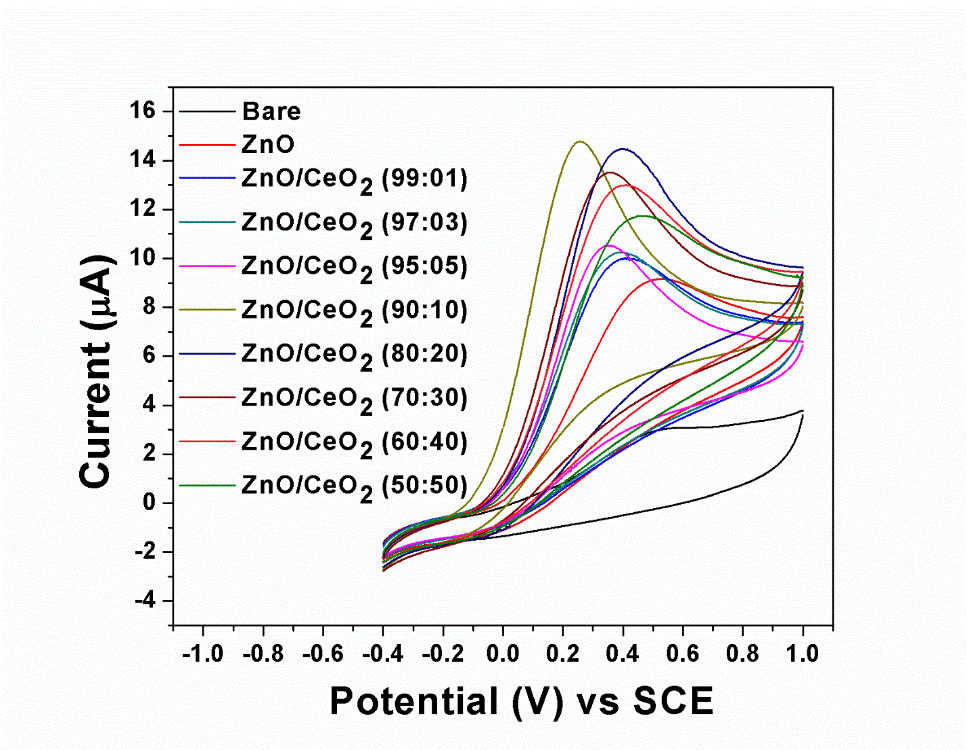
**

**Fig. S4.** The cyclic voltammetry (CV) response of 3 mM UA for bare, pure ZnO and ZnO/CeO_2_ nanocomposites at a scan rate 50 mV/s.
